# Potential of Stainless Steel Slag Waste in Manufacturing Self-Compacting Concrete

**DOI:** 10.3390/ma13092049

**Published:** 2020-04-28

**Authors:** Julia Rosales, Francisco Agrela, José Antonio Entrenas, Manuel Cabrera

**Affiliations:** Construction Engineering Area, University of Córdoba, 14071 Córdoba, Spain; jrosales@uco.es (J.R.); mc1enanj@uco.es (J.A.E.); manuel.cabrera@uco.es (M.C.)

**Keywords:** stainless steel slag, treatment, self-compacting concrete, mechanical and durability properties

## Abstract

The volume of slags generated from the steel industry is a source of possible resources which is constantly increasing. Specifically, in the production of stainless steel, specific and singular slags with unique characteristics are obtained, which allows considering an approach aimed at their use in new recycling ways. This work shows the feasibility of using stainless steel slag as a substitute for limestone filler in the manufacture of self-compacting concrete. The influence of different treatments applied to slags on physical and chemical properties was studied. On the other hand, the mechanical behaviour, as well as the durability acquired in self-compacting concrete, has been analysed. Very encouraging results were obtained, since this research demonstrates the possible application of this stainless steel slag as a construction material, improving sustainability and promoting circular economy processes, which are achieved through the minimisation of the waste disposal and accumulation.

## 1. Introduction

In the industrial and construction sectors, it is increasingly essential to introduce sustainability both in the use of new materials and raw materials and in production processes. The manufacture and production of steel materials is one of the most important industries in the world. Different raw materials, in their various industrial processes, involve the generation of different waste. The main waste generated in volume in the manufacturing processes of these materials is steel slag [1].

Steel demand and production in Europe, Asia, the Americas and China experienced moderate growth in 2018. Among the main drivers of steel demand in Europe are the construction and mechanical engineering industries [2]. 

The steel production sector includes stainless steel. The main raw materials used in the manufacture of stainless steel are nickel, ferrochrome, molybdenum and ferrous scrap.

World production of stainless steel in 2018 exceeded 50.7 million tonnes, an increase of 5.5% over the previous year’s volume. The activity of the world’s leading stainless steel producer, China, grew by 3.6% to 26.7 million tonnes. This figure represents 52.6% of world production of stainless steel [3].

Despite the fact that stainless steel is a material that can be recycled over and over again, which makes it perfectly suited to sustainable development and the circular economy, its production generates certain residues, stainless steel slag (SSs) being that generated in greater volume (78%) [4].

SSs have similar chemical properties and microstructure to other more studied steel slags such as blast furnace slag (BFS). They are composed mainly of calcium and silica oxides, and these chemical characteristics show pozzolanic properties [5]. Despite the pozzolanic potential that SSs may have due to their composition, there are few studies that research their use as a substitute for cement. Zhang et al. [6] replaced 10% cement with SSs demonstrating that crushing the material increased the reactivity and strength of the manufactured materials. Similar results were obtained by Rosales et al. [7], where a 30% cement substitution was used.

Research on the use of SSs has focused on the application of this waste as a substitute for fine and coarse aggregates in the manufacture of SCC and conventional concrete [8]. Concrete made with SSs provides better mechanical properties and durability compared to conventional concrete, despite the difficulties in mixing preparations [9]. 

The physical properties, pozzolanic characteristics and the positive results obtained in the application of SSs as a substitute for cement and aggregates in concrete lead to the study of its application in the manufacture of self-compacting concrete (SCC). SCC is a concrete that is characterised by a certain viscosity and, at the same time, differs from conventional concretes with a fluid consistency. A high percentage of fine-grained materials are used for the production of SCC, which are mainly provided by the cement, the additives and the limestone filler, which is often used in this type of concrete, applying the corresponding decrease of the proportion of coarse aggregate.

Industrial by-products such as ash, marble and glass dust, silica fume, out-of-use tyres, recycled volcanic ash and blast furnace slag are generally used as materials for the production of recycled self-compacting concrete [10,11,12,13,14,15,16]. The widespread use of these waste materials is mainly due to their particle size. They are called powdery by-products with a particle size below 0.150 mm. 

The use of steel slag in the manufacture of SCC has focused on the replacement of coarse and sand. More limited research has been conducted on its use as a substitute for cement. The use of this by-product leads to a reduction in SCC fluidity and similar mechanical strength results to SCC controls [17,18,19].

The objective of this study is to evaluate the viability of using SSs as a substitute for limestone filler in the manufacture of SCC. The SSs were processed by applying four different treatments, so that mixes were manufactured that included unprocessed (SSs), crushed and sieved (SSs-C), burned (SSs-B) and a combination of crushing and burned (SSs-CB).

The physical and chemical properties of the SSs were characterised, and a study of the mechanical behaviour and durability properties of SCC manufactured with SSs was also carried out. Compressive strength values were very similar to those obtained in the SCC control mixture. Mechanical performance was improved when SSs were treated by crushing and burning processes. In terms of durability parameters, the use of SSs leads to improved performance as a barrier to reduce chloride ion penetration, an essential feature to prevent degradation. Finally, the application of these SSs in SCC leads to a decrease in carbonation penetration and water penetration. 

## 2. Materials Characterization and Dosage

This study evaluates the feasibility of using SSs with four different processes as a substitute for limestone filler. Three series of SSs were carried out, differentiated according to the percentage of substitution and cement content. The materials used, the treatment procedure given to the SSs and the dosages studied are shown below.

### 2.1. Cement and Natural Aggregates (Aggregates, Sand and Filler)

**Figure 1:**
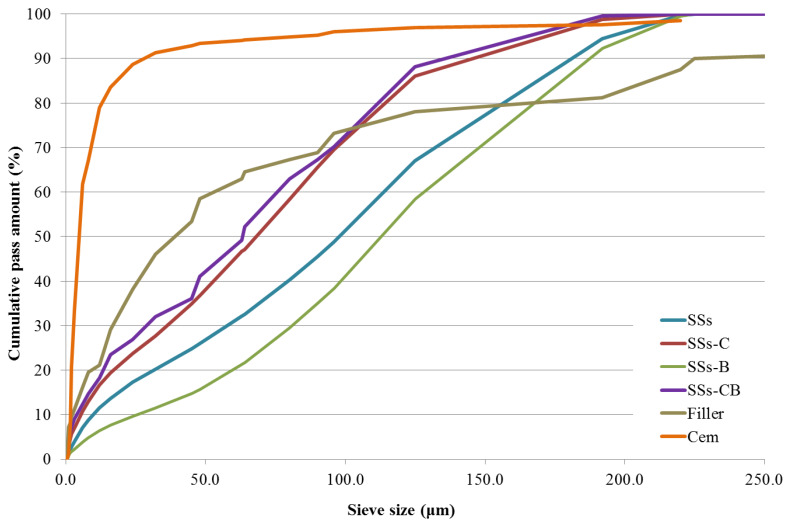
Particle size distribution of SSs and Filler.

The materials commonly used by companies producing this concrete have been used for the manufacture of SCC. Each of them and their characteristics are detailed below:

- Cement: CEM I-42.5 R, from Cementos Portland Valderrivas. This cement has high initial mechanical resistance, which makes it easy to manipulate and remove from the mould in the early stages. It is composed of 97% clinker, 3% limestone and gypsum as a setting regulator. The particle size distribution is shown in Figure 1. The cement shows a higher fineness than the rest of the materials used.

- Natural aggregates. The natural aggregates used in this work were supplied by the company Pretersa Prenavisa, a company dedicated to engineering, design and manufacture of concrete, so its raw material (sand, filler and gravel) meets the technical specifications for use in SCC. Each one of the aggregates was grouped according to its granulometry considering the fractions 4–200 mm for gravel, 0–4 mm for sand and 0–2 mm for limestone filler. Figure 1 shows the particle size distribution of the material.

### 2.2. Processed Stainless Steel Slag

The stainless steel slag (SSs) come from the company Acerinox Europa S.A.U. located in Los Barrios (Cádiz), Spain. 

**Figure 2:**
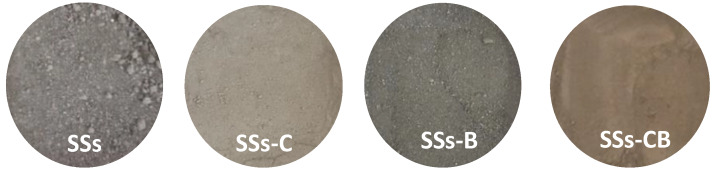
SSs treatments.

The SSs were processed in the laboratory, obtaining four different materials to be used in the manufacture of SCC (Figure 2): SSs (stainless steel slag dried in an oven for 24 h and screened with a particle size not exceeding 1 mm), SSs-C (stainless steel slag dried in an oven for 24 h and crushed, with a particle size not exceeding 0.25 mm), SSs-B (stainless steel slag burned in an oven at 800 °C for 24 h) and SSs-CB (stainless steel slag with combined crushing and burning treatment). 

A decrease in particle size was observed when the SSs were subjected to crushing and combined burning and crushing treatment; the particle size of such processed SSs was similar to the particle size of the filler (Figure 1). 

The physical properties of SSs according to different standards are shown in Table 1. SSs showed high density and absorption. Treatment of the material by crushing and burning processes reduced the density and the absorption of the material. The different test methods used are mentioned in Appendix A.

In relation to chemical properties, SSs show high SiO_2_ and CaO contents. This composition is similar to those of steel slags composed mainly of silica, calcium oxide, magnesium oxide, aluminium and iron [19,20]. 

No significant changes were observed in the elemental components of the SSs to which some treatment was applied.

Because chlorides can cause corrosion in reinforcements used in combination with structural concrete and can affect durability parameters [21], the presence of water-soluble chlorides was evaluated. The results obtained showed a decrease in the presence of chlorides (g/100 g) in all SSs samples that were processed.

The mineralogical study of the SSs was performed by powder X-ray diffraction (XRD) on a Bruker (D8 Advance A25 model) with Cu anticoat. The measurement conditions were: 2θ of 10°–120°; pitch = 0.015°; t = 0.5 s; tube conditions: 40 kV and 30 mA; fixed divergence gap of 0.1°; spin of 30 rpm and Ni filter. DifracPlus EVA 3.1 software was used for the identification of the crystalline phases, and the Rietveld method was used for the quantitative analysis by XRD (TOPAZ program v. 4.2)

**Figure 3:**
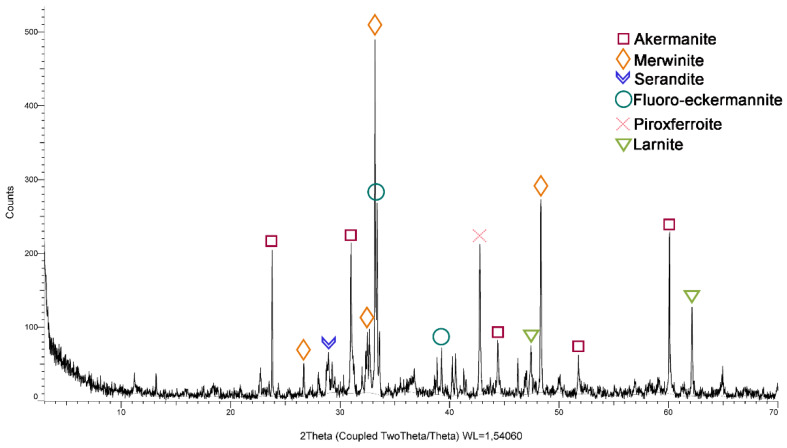
SSs mineralogical composition by X-Ray diffraction (DRX).

Figure 3 shows that SSs are mostly composed of merwinite, calcium oxide, magnesium, and silica and akermanite (Ca-Mg-Si). This mineralogy corresponds to the majority composition of SSs.

In addition to the elemental chemical composition, a study of the concentrations of the most polluting components for the environment through the leachate was carried out. This study was carried out using the UNE-EN 12457-4:2003 procedure. This is a compliance test to determine whether the waste complies with the specific reference values. 

For this procedure, the sample is diluted in a concentration with a liquid/solid ratio (L/S) equal to 10 with deionised water and is shaken by a mechanical tumbler. After the mixing time, the solids are allowed to decant, pH, conductivity and temperature are measured, and the eluate is extracted with a syringe and filtered with a membrane filter. The samples are placed in test tubes which will be analysed in an ICP (inductively coupled plasma) mass spectrometer.

Leaching analysis is focused on determining the concentrations of heavy metals regulated by the European Landfill Directive, such as As, Pb, Cd, Cr, Cu, Hg, Ni, Zn, Ca, Mg, Se and Sb. 

Table 2 shows the leaching results of SSs and each of their treatments.

The limit values are established in accordance with the standards that regulate the emission of heavy metals from accumulated waste in landfills. It should be noted that SSs will be applied within a cementitious matrix and will account for 6% of the total weight of the material used in the manufacture of SCC.

The high content of chrome and molybdenum is common in steel slag due to the raw materials used and its production system [7,22,23]. 

SSs exceeded the limits established to be considered inert material, being classified as non-hazardous because the values of chromium exceed the established limits (Table 2).

A considerable increase in Cr is observed in the samples that have been processed by calcination as compared to those that have not been processed, as occurs with the rest of the elements, Se and Mo. The SSs-B showed the highest values of these three elements.

### 2.3. Dosage

The most important condition to be considered for batching an SCC is to provide enough of the “cement + water + fine aggregates” set to achieve the self-compacting characteristics. The materials used in the manufacture of the SCC do not differ from those of conventional concrete, although it is necessary to substantially increase the amount of very fine particle size aggregates (particles that pass through the 0.125 mm sieve). It is necessary to control the water/cement ratio so as not to produce strength losses [24]. Powdered pozzolanic fillers and fluidizing agents that provide viscosity are necessary for better cohesion and stability of the mixture [25,26,27]. In addition, additives are added to obtain the required consistency and self-compacting properties [28].

Considering these preconditions, the replacement of limestone filler by SSs with different treatments has been carried out.

First the coarse aggregate, fine aggregate, filler or SSs with 50% of the total water are introduced into the mixer and mixed for five minutes. Then, the cement and the additive dissolved in the remaining water are added and homogenised for ten minutes. 

The consistency was determined in the fresh state, and test samples were produced. The specimens were demoulded after 24 h and cured in a chamber at 20 °C and 100% humidity.

This study was carried out considering the consistency and the total volume of material produced. In this way, an optimal dosage was achieved that was similar to the control dosage, replacing the limestone filler with different amounts of SSs with the different processes applied (Table 3).

Three groups of concrete, SCC-30, SCC-30 50/50 and SCC-50, were manufactured and four series of mixes were made in each of them:

SCC: Control concretes made with limestone filler.

SCC-SSs: This second series of SSC was manufactured with unprocessed SSs and controlling parameters such as humidity, density, volume, water consumption and additives.

SCC-SSs-C: Manufacture of SSC with SSs processed by crushing processes.

SCC-SSs-B: Manufacture of SSC with SSs that were burned at 800 °C.

SCC-SSs-CB: Manufacture of SSC with SSs that were burned and crushed.

## 3. Testing Methods and Results

This study evaluated the physical, technical and durability properties of SCC in the fresh and hardened states. Table 4 shows each of the tests carried out, the standards applied and the age of the test specimens.

### 3.1. Flowability

A detailed study of the consistency of the fresh concrete is essential as it is a basic characteristic for the production of SCC [29]. Consistency was evaluated by three different methods, slump test, L-box and J-ring. Table 5 shows the results obtained.

The results obtained from the three consistency measurement methods are within the limits established for an SCC, considering that the use of SSs in the manufacture of SCC gives the concrete low viscosity behaviour. The use of other types of slag such as fly ash or rice husks generally leads to a negative effect on the consistency of the SCC [30]. This effect was not observed with the use of SSs (Table 5).

An increase in slump flow was observed for mixtures in which SSs were applied with crushing and burning treatment. The use of SSs without any treatment reduced slump flow with respect to the control. Similar results were obtained for the L-box test. However, the J-ring test showed a lower slump flow value for concretes manufactured with SSs.

### 3.2. Compressive Strength. 

A study of the compressive strength over time of each of the SCC series manufactured was carried out. Table 6 shows a significant increase in the resistance values as the age of the manufactured concretes has increased, the average of the strength at 7 days being of 59% of the strength acquired at 28 days for the SCC-30 and SCC-30 series (50/50). In the case of the SCC-50 series, its strength at 7 days increases to 79% of the strength at 28 days.

All the series produced with SSs presented similar or higher results than those obtained in the control series, manufactured with natural aggregates (Table 6).

The improvement in compressive strength may be due to the pozzolanic activity of these residues, as was found in previous studies using coal ash [31].

The use of by-products or recycled aggregates as a substitute for the fine fraction directly affects the compressive strength, resulting in a reduction with respect to SCC made from natural aggregates [32,33]. Most of the previous works are focused on the replacement of the coarse and fine fraction of the natural aggregates, obtaining optimum replacement ranges depending on the type of industrial by-product used, around 15%–50% [31,34,35]. 

In this work, a range of 100% limestone filler substitution was proposed, an essential element to avoid SCC segregation. The use of SSs provided the mixture with sufficient cohesion, workability and compressive strength.

**Figure 4:**
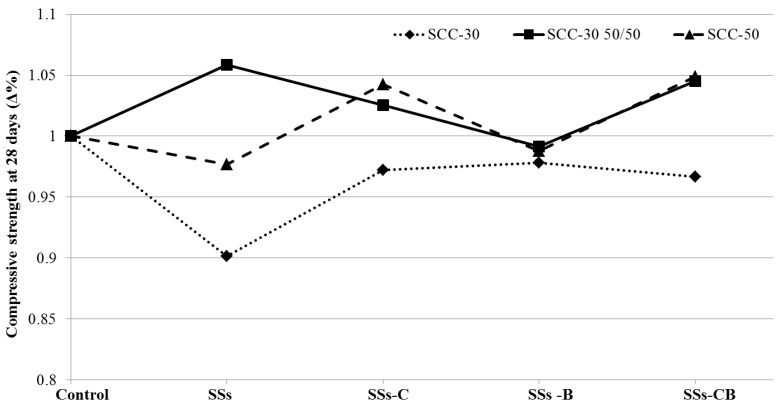
Relative compressive strength of SCC-30, SCC-30 50/50 and SCC-50 at 28 days versus control.

The treatment of the SSs showed an improvement in the results of compressive strength, being the crushing treatment one of the most effective in the three series manufactured as shown in Figure 4.

The 28-day compressive strength analysis showed for the SCC-30 series a 10% reduction for the use of untreated SSs. When SSs were processed, the compressive strength was reduced by approximately 2.5% over the control. Similar behaviour was observed for the SCC-50 series; however, in this case, the loss of strength for the use of SSs was only 2%. The use of SSs-C and SSs-CB resulted in an increase of approximately 4.5% in compressive strength at 28 days (Figure 4).

### 3.3. Tensile Splitting Strength

**Figure 5:**
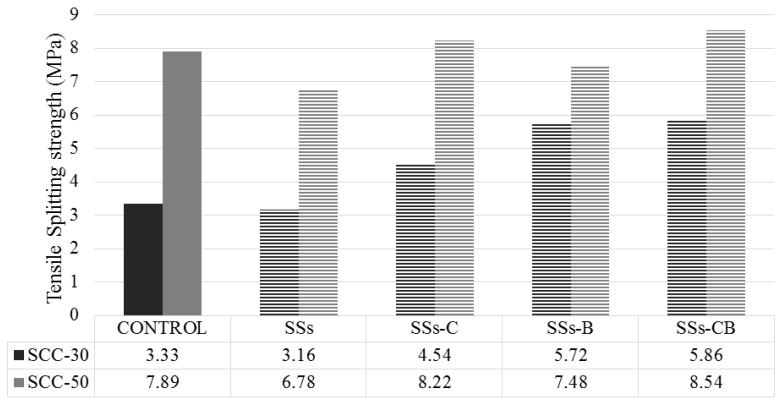
Tensile Splitting strength of SCC-30 and SCC-50.

A 28-day tensile strength study of the SCC-30 and SCC-50 series was carried out as 100% of the filler replacement was applied, and the compressive strength results were positive. The results obtained are shown in Figure 5.

Tensile splitting strength values were observed in the concrete representing approximately 10%–15% of the results obtained for compressive strength. The increase in material density is associated with higher values of tensile strength [36].

It was observed that the 28-day tensile strength of the SCC-50 series was higher than that of the SCC-30.

The use of SSs as a substitute for limestone filler in the manufacture of SCC resulted in an increase in the splitting tensile values when the SSs were subjected to some type of treatment, obtaining values greater than the control. The highest value was obtained in concretes manufactured with SSs-CB for both series. 

### 3.4. Shrinkage

A study of the dimensional changes produced in the SCC was carried out under two different curing conditions, underwater curing (humidity 100%, temperature 20 °C) (UW) and dry chamber curing (humidity 50%, temperature 20 °C) (DC).

**Figure 6:**
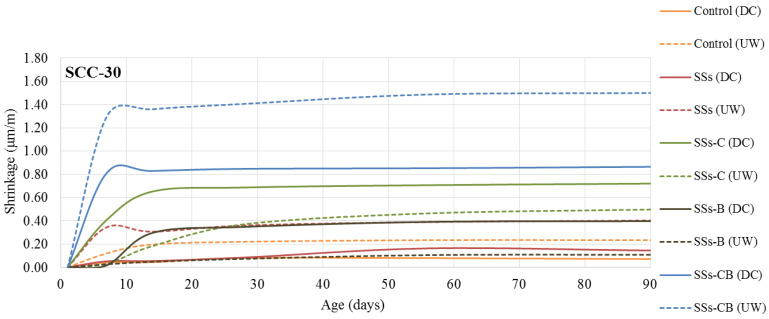
Shrinkage of the SCC-30 as a function of age in days. (UW) Underwater curing condition, (DC) Dry Chamber curing conditions.

**Figure 7:**
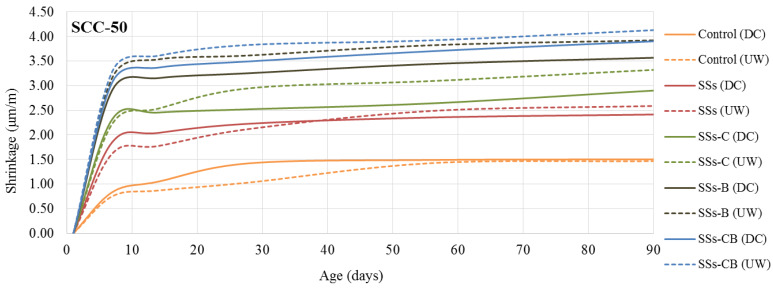
Shrinkage of the SCC-50 as a function of age in days. (UW) Underwater curing condition, (DC) Dry Chamber curing conditions.

Figure 6 and Figure 7 show the results obtained for the SCC-30 and SCC-50 series, respectively, over time, with measurements taken up to 90 days. The maximum shrinkage values established by Spanish concrete standards [37] show values much higher than those obtained. In SCC-30, the regulations establish maximum shrinkage values of 75 μm/m for submerged cured concretes and 150 μm/m for concrete cured in a dry chamber at 90 days. All the SCC-30 manufactured showed shrinkage much lower than these values. The same occurs with the SCC-50 values, in which the regulations establish as maximum shrinkage at 90 days values of 112 μm/m and 173 μm/m in underwater cured and dry chamber conditions, respectively.

In contrast to previous studies which showed that variation in limestone filler content does not affect SCC [38], the use of SSs led to an increase in shrinkage values compared to the control.

The figures show greater dimensional changes in SCC-50 than in SCC-30, mainly due to the higher cement content [39]. 

In relation to the curing conditions, SCC underwater-cured (UW), for both series, generally presented greater shrinkage than dry chamber-cured (DC) concrete.

The lower shrinkage values, similar to the control, are observed with the use of SSs and SSs-C in the SCC-30 series. The use of SSs in the SCC-50 series led to an increase in shrinkage over control concrete. 

The fineness of the slag has an important influence on shrinkage at short ages [40]. It has been proven that the crushing of the slag leads to an increase in the shrinkage values. The combination of crushing and calcination was the treatment of the SSs that led to a higher shrinkage in both concrete series 

### 3.5. Water Penetration under Pressure

According to the regulations set out in Table 4, a study of water penetration under pressure at the age of 28 days was carried out, assuming that at this age the SCC is completely cured and its durability can be evaluated.

According to previous studies, the binder content and the water/cement ratio is directly dependent on water absorption. The higher the water/cement ratio increase the greater the pore volume and thus the absorption [41,42]. The type of material used as a substitute for the coarse fraction also played a role. If the by-product or waste used has a high porosity, it will influence the water absorption in the SCC mixture so it will be necessary to use a fluidizing agent to achieve equal workability and not to influence the mechanical and durability properties of the SCC [16,43,44].

**Figure 8:**
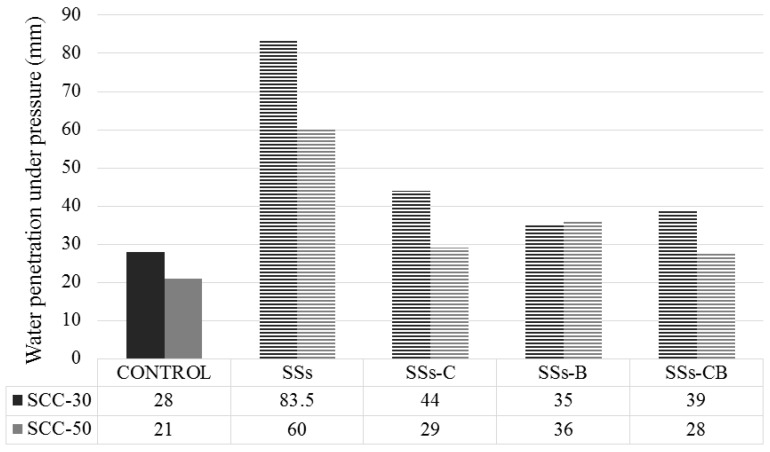
Water penetration under pressure at 28 days of SCC-30 and SCC-50.

Similar results were obtained in this work. The SCC-50 series with a lower w/c ratio showed less water penetration than the SCC-30 series (Figure 8). 

Figure 8 shows that the highest values of water penetration correspond to concretes manufactured with unprocessed SSs in both series, mainly due to the larger particle size.

Water penetration decreased when SSs were treated. One of the main reasons for this, besides the decrease in particle size, is a decrease in porosity as a result of the pozzolanic activity that occurs in the microstructure of the hardened cement when Ca(OH)_2_ is transformed into extra hydrated calcium silicate [45]. SSs with some type of treatment showed a higher CaO value (Table 1), which led to increased pozzolanic activity and reduced porosity.

### 3.6. Chloride Ion Penetration

Chloride penetration is a determining property in the life of buildings built with reinforced concrete structures. This process of penetration of the chloride ion into the interior of the concrete, favoured by an increase in its porosity, is produced by diffusion of the chloride ion solution in the pores of the concrete, moving towards the interior of the mass and seeking new channels of penetration. It is therefore necessary to determine the diffusion of chlorides in the different mixes made, to assess their suitability for this property. The diffusion of the chloride ion was determined by the ASTM C1202 method, through which the electrical conductivity was determined as an indirect measure of penetration. For this process, specimens with a radius of 150 mm and generatrix of 300 mm were used and extracted from the same sections cut to a thickness of 50 mm. After waterproofing with epoxy resin on the perpendicular side of the bases and immersing the specimens in water under vacuum for 18 h, two electrodes were attached to each specimen. An oscilloscope measured the conductivity for 6 h.

**Figure 9:**
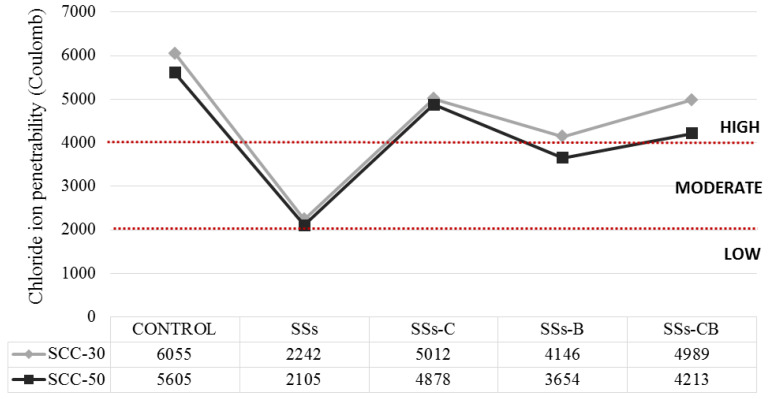
Chloride ion penetration test at 28 days of series SCC-30 and SCC-50.

Figure 9 shows the values obtained. It can be seen that lower chloride diffusion values were obtained in the concretes in which SSs were used.

Similar results were obtained in previous studies, which showed that the use of by-products such as silica fume or fly ash significantly reduced chloride ion penetration, demonstrating greater resistance than a control SCC [46,47].

It is remarkable that all the concretes manufactured with SSs can be classified as moderate or with values very close to the limit established as high, unlike the control concrete that presented high values of chloride ion penetration (Figure 9).

The concretes made with unprocessed SSs presented the best results, and despite the fact that the samples of these concretes resulted in a higher porosity than the control concrete; however, in a somewhat contradictory way, the diffusion of chlorides is reduced in this case. 

**Figure 10:**
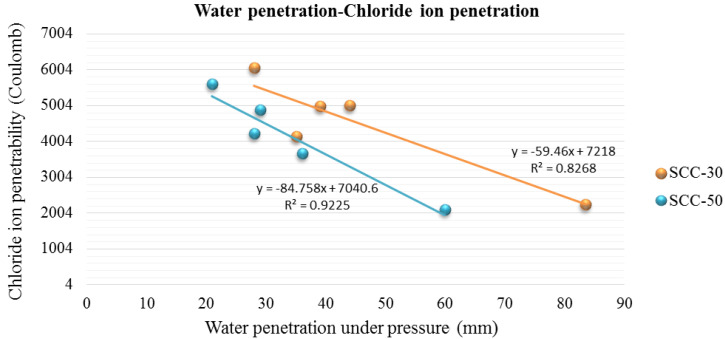
Water penetration under pressure-Chloride ion penetration of series SCC-30 and SCC-50.

Unlike water penetration, chloride penetration decreased with the application of SSs. Both values are related to each other, as can be seen in Figure 10.

Concrete made with SSs has higher porosity than control concrete, which leads to higher water penetration and shrinkage values; however, it reduces chloride conduction, a fundamental characteristic for the use of SCC.

### 3.7. Carbonation Depth

Carbonation is the phenomenon that occurs when CO_2_ comes into contact with the portlandite generated during the curing of concrete. During this process, calcium carbonate is formed in the presence of water. The problem associated with carbonation is a loss of passivity of the reinforcement and therefore an increase in the possibility of corrosion, mainly due to a reduction in the pH. 

**Figure 11:**
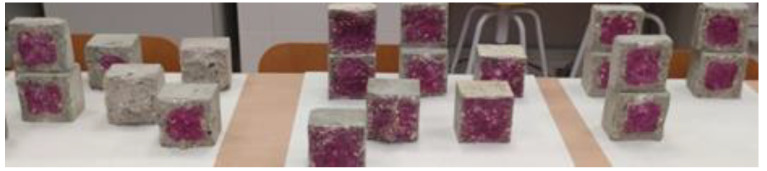
Carbonated SCC probes.

The UNE 112011:2011 method was used to determine the penetration of carbonates. The test was conducted in an accelerated carbonation chamber (60% relative humidity at 23 °C) with 5% of CO_2_, in prismatic specimens measuring 100 × 100 mm, cured for 28 days in a humid chamber. The depth of carbonation was measured at 1, 28 and 56 days (Figure 11).

The use of SSs in the manufacture of SCC showed an increase in carbonation depth for all series. The use of SSs-C reduced this depth. 

**Figure 12:**
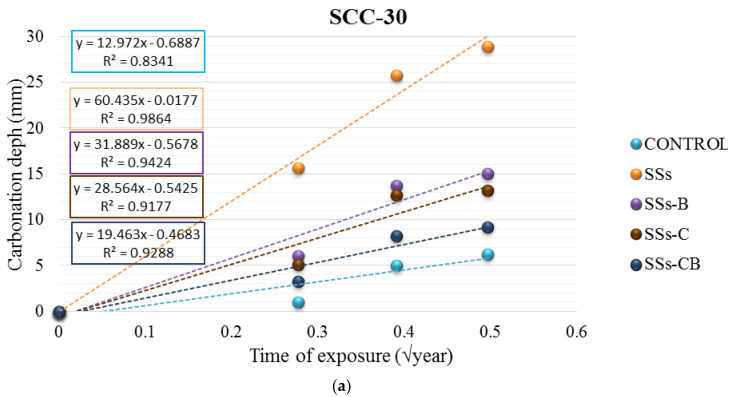

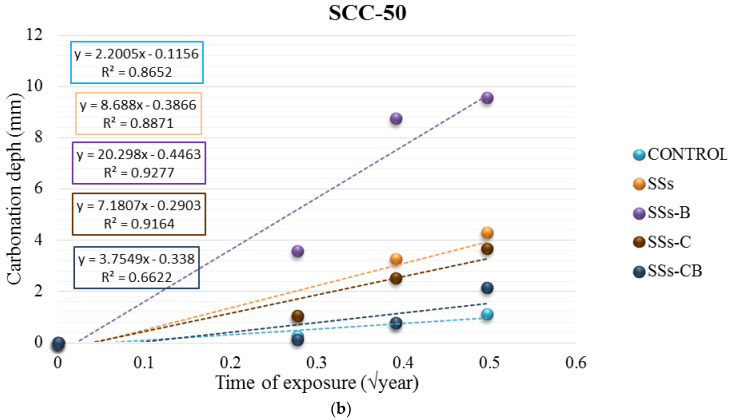
Depth of carbonation according to √*t* of SCC-30 (**a**) and SCC-50 (**b**).

The carbonation produced in the material was also evaluated from the carbonation coefficient. Figure 12 shows the depth of carbonation (Cd) compared to the square root of time (years). 

The highest carbonation coefficient was produced in SCC-30 with the use of raw SSs (60.435 mm/√year). In the SCC-50 series, the highest values were obtained in the mixtures in which SSs-B were applied (20.298 mm/√year).

These results can be seen from the point of view of material porosity and curing reactions.

## 4. Conclusions

This study has evaluated the properties of SSs with different treatments and the influence that its use has on the mechanical and durability properties in the manufacture of SCC.

The following conclusions were obtained:

- SSs present an elemental composition of SiO_2_, CaO and MgO. The mineralogy of the SSs showed high levels of merwinite, calcium oxide, magnesium, silica and akermanite (Ca-Mg-Si). The treatment of crushing and burning of SSs leads to an increase in SiO_2_ and CaO values, as well as a decrease in density and absorption.
- The environmental study by means of leaching classifies SSs as non-hazardous material due to the high values of Cr. The burning treatment increases the leaching values of these elements, Se and Mo.
- In relation to the mechanical properties for the SCC-30 series, the compressive strength of SCC manufactured with SSs decreases slightly with respect to the control. The burning and crushing treatment of the SSs improves the mechanical properties. In the SCC-50 series, higher compression values are obtained with the use of SSs. The treatment in this case leads to a reduction in the values.
- In terms of durability parameters, SCC-30 has smaller dimensional changes than SCC-50 has. The use of SSs in the manufactured of SCC results in greater dimensional instability, especially when SSs are processed. In relation to chloride ion penetration, a reduction of this parameter can be observed in SCC manufactured with SSs as compared to control. The depth of carbonation is lower in SCC when SSs were subjected to crushing and combined crushing and burning processes.

In conclusion, the burning process of SSs remarkably improves the resulting concrete properties, such as chloride ion penetration resistance as well as water penetration under pressure resistance; however, it generates extra costs. We can consider that the application of SSs subjected to drying processes and achieving humidity lower than 4% and crushing process are the most viable. Due to the fact that both processes do not involve great costs or associated risks, it is possible to obtain adequate values of mechanical behaviour and durability properties in the manufacture of SCC with SSs as a substitute for limestone filler.

For these reasons, and looking to future lines, the application of SSs in full-scale constructions is interesting. Thus, the application of SCC with SSs in construction elements subject to real and long-term climatic conditions could be evaluated.

## Figures and Tables

**Table 1:** Physical and chemical properties of SSs.

		SSs	SSs-C	SSs-B	SSs-CB	Test Method
**Physical Properties**		-			-	-
Density-SSD (kg/dm^3^)		2.06	1.8	1.69	1.66	UNE - EN 1097-6:2014
Absorption (%)		6.12	5.31	5.44	5.41	
**Chemical Properties**		-			-	-
Elemental Content (%)	SiO_2_	28.30	29.88	29.04	30.59	UNE-EN 196-2:2014
	Al_2_O_3_	5.64	5.73	5.64	5.86	
	Fe_2_O_3_	3.09	1.31	2.30	1.17	
	CaO	42.09	44.51	43.11	44.73	
	MgO	10.97	11.68	11.22	11.63	
	SO_3_	0.39	0.34	0.33	0.35	
Water-Soluble Chlorides (%)		0.025	0.017	0.016	0.02	UNE-EN 1744-1:2010

**Table 2:** Concentrations of metals on leachate at L/S = 10 L/kg and classification according to concentration on heavy metals.

	Inert Limit - Non-Hazardous (mg/kg M.S.)	SSs	SSs-C	SSs-B	SSs-CB
		(mg/kg)	(mg/kg)	(mg/kg)	(mg/kg)
Cr	0.5–10	**2.356**	**1.328**	**6.568**	**2.190**
Ni	0.4–10	0	0	0	0
Cu	2–50	0	0	0	0
Zn	4–50	0.003	0	0	0
As	0.5–2	0.002	0	0	0
Se	0.1–0.5	0.097	0.089	**0.171**	0.048
Mo	0.5–10	0.463	0.486	**1.602**	0.288
Cd	0.04–1	0	0	0	0
Sb	0.06–0.7	0.017	0.001	0.001	0.001
Ba	20–100	1.776	0.294	7.790	6.642
Hg	0.01–0.2	0	0	0	0
Pb	0.5–10	0	0	0	0

Inert value limits exceeded are given in bold and underlined.

**Table 3:** Mix proportion (kg/m^3^).

		Sand	Aggregates	Cement	Filler	Additive	Water	W/C Relation	SSs			
		(0–4 mm)	(4–200 mm)	-	-	-	-	-	SSs	SSs-C	SSs-B	SSs-CB
SCC-30	SCC-30-Control	1000	700	325	125	3.41	195	0.60	-	-	-	-
	SCC-30-SSs	1000	700	330	-	3.3	210	0.64	115	-	-	-
	SCC-30-SSs-C	1000	700	325	-	3.25	205	0.63	-	125	-	-
	SCC-30-SSs-B	1000	700	325	-	3.25	205	0.63	-	-	125	-
	SCC-30-SSs-CB	1000	700	325	-	3.25	205	0.63	-	-	-	125
SCC-30 50/50	SCC-30-0.5SSs	1000	700	330	63	3.30	201	0.61	58	-	-	-
	SCC-30-0.5SSs-C	1000	700	325	63	3.25	198	0.61	-	63	-	-
	SCC-30-0.5SSs-B	1000	700	325	63	3.25	198	0.61	-	-	63	-
	SCC-30-0.5SSs-CB	1000	700	325	63	3.25	198	0.61	-	-	-	63
SCC-50	SCC-50-Control	1000	700	450	20	4.95	180	0.40	-	-	-	-
	SCC-50-SSs	1000	700	450	-	4.90	185	0.41	20	-	-	-
	SCC50-SSs-C	1000	700	450	-	4.90	185	0.41	-	20	-	-
	SCC50-SSs-B	1000	700	450	-	4.90	185	0.41	-	-	20	
	SCC50-SSs-CB	1000	700	450	-	4.90	185	0.41	-	-	-	20

**Table 4:** Test Methods.

		Test Method	Curing Time
Technological tests	**Properties of Fresh SCC**	-	-
	Flowability	UNE-EN 12350-8:2011 UNE-EN 12350-10:2011 UNE 12350-12:2011	0 days
	**Properties of Hardened SCC**	-	-
	Compressive strength	UNE 12390-3:2009	1, 7, 28, 56, 90 days
	Tensile splitting strength	UNE-EN 12390-6:2010	28 days
	Shrinkage	ASTM C157-17	1, 14, 28, 56, 90 days
Durability properties	Water penetration under pressure	UNE 12390-8:2009	28 days
	Chloride Ion Penetration	ASTM C1202-97	28 days
	Depth carbonation	UNE 112011:2011	28, 56, 90 days

**Table 5:** Fresh properties of SCC.

		Slump Test	L-Box Test			J-Ring
		D (mm)	T_200_ (s)	T_400_ (s)	H_2_/H_1_ (%)	DH = H_1_ − H_2_ (mm)
Compliance Requirements		650–750	<1.5	<2.5	0.8–1	<10
(Recommended)						
SCC-30	SCC-30-Control	712	1.23	1.97	0.88	5.1
	SCC-30-SSs	706	1.12	1.86	0.87	4.3
	SCC-30-SSs-C	725	1.67	2.13	0.91	4.2
	SCC-30-SSs-B	718	1.56	2.01	0.89	4.1
	SCC-30-SSs-CB	719	1.64	2.08	0.89	4.1
SCC-30 50/50	SCC-30-0.5SSs	711	1.33	2.25	0.87	5.02
	SCC-30-0.5SSs-C	719	1.93	2.01	0.91	4.7
	SCC-30-0.5SSs-B	703	1.21	2.14	0.87	4.6
	SCC-30-0.5SSs-CB	716	1.38	2.23	0.9	4.6
SCC-50	SCC-50-Control	726	2.05	1.98	0.91	4.5
	SCC-50-SSs	732	2.11	2.29	0.91	4.01
	SCC50-SSs-C	748	2.21	2.36	0.93	3.9
	SCC50-SSs-B	744	2.13	2.33	0.93	4.1
	SCC50-SSs-CB	741	2.18	2.44	0.92	4.2

**Table 6:** Compressive strength of SCC.

		Compressive Strength (MPa)				
		Days				
		1	7	28	56	90
SCC-30	SCC-30-Control	8.39	25.86	42.21	42.36	46.55
	SCC-30-SSs	5.12	21.89	38.05	41.03	42.54
	SCC-30-SSs-C	9.48	26.08	41.03	42.15	43.21
	SCC-30-SSs-B	10.04	28.52	41.29	43.28	44.36
	SCC-30-SSs-CB	9.81	24.15	40.81	41.35	42.77
SCC-30 50/50	SCC-30-0.5SSs	10.12	29.15	44.68	45.28	47.44
	SCC-30-0.5SSs-C	9.15	29.66	43.28	47.16	48.56
	SCC-30-0.5SSs-B	8.66	27.38	41.85	43.06	44.73
	SCC-30-0.5SSs-CB	9.12	30.06	44.12	48.27	48.98
SCC-50	SCC-50-Control	18.39	53.38	63.14	65.26	66.87
	SCC-50-SSs	19.85	44.25	61.69	64.11	65.28
	SCC50-SSs-C	21.02	58.3	65.83	68.91	69.33
	SCC50-SSs-B	20.35	46.24	62.38	65.87	67.05
	SCC50-SSs-CB	23.87	61.79	66.22	69.06	70.15

## Appendix A

Standards used in the experimental work:

UNE-EN 1097-6:2014. Tests for mechanical and physical properties of aggregates—Part 6: Determination of particle density and water absorption

UNE-EN 196-2:2014. Method of testing cement—Part 2: Chemical analysis of cement

UNE-EN 1744-1:2010. Tests for chemical properties of aggregates—Part 1: Chemical analysis

UNE-EN 12457-4:2003. Characterisation of waste - Leaching - Compliance test for leaching of granular waste materials and sludges—Part 4: One stage batch test at a liquid to solid ratio of 10 l/kg for materials with particle size below 10 mm (without or with size reduction)

UNE-EN 12350-8:2011. Testing fresh concrete—Part 8: Self-compacting concrete - Slump-flow test

UNE-EN 12350-10:2011. Testing fresh concrete—Part 10: Self-compacting concrete - L box test

UNE 12350-12:2011. Testing fresh concrete—Part 12: Self-compacting concrete - J-ring test

UNE 12390-3:2009. Testing hardened concrete—Part 3: Compressive strength of test specimens

UNE 83306-85. Testing hardened concrete—Part 6: Tensile splitting strength of test specimens

ASTM C157-17. Standard Test Method for Length Change of Hardened Hydraulic-Cement Mortar and Concrete

UNE 12390-8:2009. Testing hardened concrete—Part 8: Depth of penetration of water under pressure.

ASTM C1202-97. Standard Test Method for Electrical Indication of Concrete’s Ability to Resist Chloride Ion Penetration

UNE 112011:2011. Corrosion of concrete reinforcement steel. Determination of the carbonatation depht for in-service concrete.
